# Rigidity of the Os Uteri Overcome in Obstetric Practice

**Published:** 1876-09

**Authors:** 


### RIGIDITY OF THE OS UTERI OVERCOME IN OBSTETRIC PRACTICE.

I was deeply interested in an able article from the pen of Philip Adolphus, M.D., in the April number of the Chicago *Medical Journal and Examiner*, on the "Relative Position of Chloroform, Sulphuric Ether and Sulphate of Morphia in Obstetric Practice."

For eleven years, obstetrics and diseases of women have been almost a specialty in my practice; and for the past two years I have entirely discontinued the use of chloroform, with one excptional case, considering chloral hydrate in every way preferable. Taken in combination with Doveri pulv., or liq. Doveri, I have overcome the (apparently) most obstinate cases of rigidity of the os uteri.

The exceptional case, in which I tried to administer chloroform, was that of a lady twenty-seven years of age, pregnant with her third child. The husband came in great haste, saying his wife was flowing to death, and was delirious, with incessant pain. I had attended the lady in both her previous confinements, and she had no unusual amount of suffering nor abnormal symptoms. At this time I found her deliirous and in irregular spasms. Attempted the administration of chloroform, but, owing to the dusky appearance of the countenance and impeded respiration, had to desist. On digital examination, found os uteri rigid and dilated, but sufficient to admit tip of index finger. With every pain, which was as often as three, and two and a half minutes, was a profuse gush of blood. Gave dr. j of Squibb's fld. ex. ergota, chloral hydr, grs. x. In fifteen minutes the pains were less convulsive and frequent, and flow greatly moderated. But the rigidity of os remaining the same, and pains occurring every five minutes, with no perceptible increase of dilation, gave Dover's pulv. in connection with chloral hydr. I have *never* failed, in using this remedy, and think it preferable to morphia, as consciousness and sensibility are not destroyed, and it has the relaxing properties of the ipecac. After giving two doses of the following formula—chloral hydrate, dr. j; liq. Doveri, dr. jss; syr. cinn. oz. j; M. sig., a teaspoonful at intervals of thirty minutes, the attendants ceased controlling the lady's movements, and she broke out into a profuse perspiration, at the same time quietly sleeping between the pains. In one hour, gave the third and last dose (I rarely give over two). In twenty minutes following, after a pain from which she indifferently roused, she said, "*There*, Dr." I slipped my hand under the sheet and found on the bed a five and a half months' fœtus, with membranes entire and unruptured, and lady had not changed her positton, and continued to sleep sweetly. She made a good recovery, had abundance of milk without any febrile symptoms.

In former years, found ipecac pulv. grs. ij. every twenty minutes, good in relaxing the rigidity of os uteri, especially in primpara labors; but it failed in allaying the irritability and extreme restlessness attending such cases. Even when administered with chloral hydrate, it has not the happy effect that Dover's powder has. For convenience the liq. Doveri (m. j, being equal to gr. j pulv.) is to be preferred. Where there are no spasmodic sypmtoms, the Dover's powder is sufficient *without the chloral*, and I prefer to use it so; and in using it have no occasion for ergota, except to control hemorrhage, *i. e.*, in uncomplicated rigidity of os uterus. If pains are *inefficient*, I substitute quinia for ergota. And in using the chloral hydr. and liq. Doveri combined, have not needed instrumental assistance, on an average once in five hundred cases—in fact, never with a normal pelvis.

Was called to a primpara labor, as was supposed. Lady in spasms, face contorted and tongue filling the mouth, which was partly open and drawn to one side. Administered dr. ij of pres. per rectum. Examined and found os rigid with no dilation perceptible. In twenty minutes the lady called for a drink of water, and seeing me, said, "Dr., am I going to be sick?" I inquired as to pain—she had no pain, bowels free, appetite good, but could not sleep. Ordered the prescription every four hours, with warm foot bath. She continued the medicine for seven days, saying, "As long as I take the medicine I am all right, but if I go over the four hours, the spells come on." At the expiration of seven days was sent for—she met me smiling, and said, "I feel some pain, but not very bad, in my back." In one hour she was delivered of a fine boy, average weight, and her first expression was, "I was not *much* sick after all." Continued the medicine three days—made a good recovery.

It is unnecessary to relate instances, but the fact is before me all the time, not only in parturition, but in my office practice, for the advantages of exploration, removal of polypi or other uterine tumors, the relaxing and sustaining properties of Dover's powder, with or without chloral hydr., as the case requires, are almost indispensible. It does not interfere with the appetite, and seldom with digestion, and its relaxing properties extend to the sphincter vaginæ, and with the usual support *I never have had a lacerated perinæum.* And there is no excitement, congestion nor increased circulation, but labor is made endurable, shorter, safer and more satisfactory. After-pains are controlled, and no complications of inflammations or lacerations as a sequel. Idiosyncrasy precluding opium, or Morphia, will accept gratefully DOVER'S POWDER.—*Chicago Medical Journal and Examiner*, August, 1876.

